# High Adherence Is Necessary to Realize Health Gains from Water Quality Interventions

**DOI:** 10.1371/journal.pone.0036735

**Published:** 2012-05-07

**Authors:** Joe Brown, Thomas Clasen

**Affiliations:** Department of Disease Control, Faculty of Infectious and Tropical Diseases, London School of Hygiene and Tropical Medicine, London, United Kingdom; University of Calgary & ProvLab Alberta, Canada

## Abstract

**Background:**

Safe drinking water is critical for health. Household water treatment (HWT) has been recommended for improving access to potable water where existing sources are unsafe. Reports of low adherence to HWT may limit the usefulness of this approach, however.

**Methods and Findings:**

We constructed a quantitative microbial risk model to predict gains in health attributable to water quality interventions based on a range of assumptions about pre-treatment water quality; treatment effectiveness in reducing bacteria, viruses, and protozoan parasites; adherence to treatment interventions; volume of water consumed per person per day; and other variables. According to mean estimates, greater than 500 DALYs may be averted per 100,000 person-years with increased access to safe water, assuming moderately poor pre-treatment water quality that is a source of risk and high treatment adherence (>90% of water consumed is treated). A decline in adherence from 100% to 90% reduces predicted health gains by up to 96%, with sharpest declines when pre-treatment water quality is of higher risk.

**Conclusions:**

Results suggest that high adherence is essential in order to realize potential health gains from HWT.

## Introduction

Over 780 million people now lack access to an “improved” water source [1], and one study has estimated the number of people who rely on microbiologically or chemically unsafe water to be 1.8 billion, or about 28% of the global population [2]. Unsafe drinking water is a leading cause of preventable disease, with the burden borne primarily by children in low and middle-income countries. Pathogens transmitted in drinking water account for an unknown but presumed significant percentage of the estimated 4 billion cases and 1.9 million deaths from diarrhoeal diseases each year [3], [4]. According to WHO and UNICEF monitoring data from 2004, diarrhoeal diseases are the second most common cause of deaths among children under 5 years of age worldwide, or 16% of the total [5].

Providing safe, reliable, piped-in water to every household is an essential goal, yielding optimal health gains while contributing to the Millennium Development Goal targets for poverty reduction, nutrition, childhood survival, school attendance, gender equity and environmental sustainability. Recent research has shown, however, that even such improved water supplies are often subject to faecal contamination [2] and that even occasional interruptions in the quality of water from piped water supplies can undermine the health benefits from safe drinking water [6]. While careful not to encourage diversion of resources away from expansion of safe piped water, public health officials have called for alternative approaches that may provide some of the health benefits of safe drinking-water as progress is made in improving infrastructure services [7].

One such alternative is household water treatment and safe storage (HWT) [8]. In many settings, both rural and urban, populations may have access to sufficient quantities of water, but that water may be unsafe for consumption as a result of microbial or chemical contamination. Effective HWT, such as boiling, filtration and chlorination, has been shown significantly improve microbial water quality [9], [10], [11], [12]. Moreover, by focusing on the point of use rather than the point of delivery, treating water at the household level minimizes the risk of recontamination [13] and the risk of contamination associated with poorly functioning or intermittent water supply systems. There is evidence that HWT can be effective in preventing self-reported diarrhoeal disease [14], [15], [16], [17], although systematic reviews have noted that the limited number of placebo-controlled trials have not reported a statistically significant reduction in risk and estimates of effect from unblinded trials may be subject to a number of well-known biases [15], [18]. Like other environmental health interventions such as sanitation or hygiene, blinding intervention studies of HWT has proved to be challenging, but additional blinded trials may be useful in evaluating the evidence for this strategy of safe water provision.

One of the other challenges that promoters of HWT have reported is lack of adherence—*correct, consistent and sustained use*, sometimes referred to as *compliance*. Unlike centrally treated, piped-in water supplies, HWT is normally a batch process that must be undertaken by the end users on a daily or other frequent basis in order to provide consistent protection against waterborne pathogens. A systematic review of chlorine-based HWT, where adherence can be objectively assessed by residual free chlorine in treated water, found several studies with adherence under 70% [14]. Even when uptake of an HWT filter intervention was high when measured by current use, 83% of adults and 95% of children were reported to also consume untreated water [19]. A number of other studies of HWT reported reduced use of interventions over time, raising questions about whether low adherence may limit the usefulness of HWT as a strategy for securing access to safe water [19], [20], [21], [22], [23], [24].

To date, however, there is limited evidence characterizing the impact of adherence on health gains attributable to HWT. A systematic review of water quality interventions to prevent diarrhoea reported a pooled risk ratio of 0.46 (95% CI: 0.25–0.84) among 16 trials reporting adherence over 50%, and 0.75 (95% CI: 0.63–0.90) among 5 trials reporting adherence of less than 50% [14]. In a review of chlorine interventions, Arnold and Colford (2007) found that the reduction of diarrhoeal disease was greater in studies that reported a higher percentage of samples with detectable levels of chlorine at the point of use [14]. Despite the limited evidence from reviews, the relationship between adherence and health impact has not been clearly characterised in field trials [16]. This is partly due to the difficulty of measuring adherence in a field setting for many technologies, especially at the level of the individual. Adherence has not been measured or reported in the majority of field trials of HWT to date.

In this paper, we present a basic quantitative microbial risk model examining the influence of pre-treatment water quality, treatment effectiveness, and adherence on predicted health gains. We seek to estimate the magnitude of the effect of variable adherence on health impacts achievable through safe water interventions, particularly HWT.

## Methods

We constructed a quantitative microbial risk assessment (QMRA) model using assumptions for ranges of untreated and treated water quality, published dose-response relationships for reference pathogens, per-case severity weighting, population susceptibility, adherence, and per-capita consumption of drinking water. In most cases, we used the default assumptions and methods articulated in and recommended by the World Health Organization (WHO)'s *Guidelines for Drinking-water Quality (GDWQ)*, 4^th^ Edition [25], where the rationale, caveats, and methodological issues that apply to this approach to estimating health risks are described in greater detail. We briefly describe the method here and provide a summary of calculations.

We report model output in disability-adjusted life years (DALYs) averted per 100,000 people per year attributable to HWT under the conditions we describe. DALYs can be used to compare health outcomes using severity and duration estimates of disease(s), combining quality and quantity of life factors [25], [26]. This measure has been extensively described previously [27] and is used here for ease of comparison with other health impact estimates.

### Reference pathogens

We used *Campylobacter jejuni*, rotavirus, and *Cryptosporidium* as reference pathogens in this risk assessment because of their relatively low infectious doses, high per-case DALY severity weighting, and the fact that we have published dose-response models for them [25]. The DALY estimates we calculated were intended to estimate the DALY contributions from bacteria, viruses, and protozoa (as classes of pathogens) that may be found in untreated water, following the approach of the WHO recommended model [25]. It is important to note that the use of these specific microbes is not a statement about their relative importance as aetiologic agents of diarrhoeal diseases globally, or their importance as specifically waterborne pathogens. Rotavirus, for example, is thought to be more frequently transmitted via pathways other than drinking water. They are all known and relatively common causes of gastrointestinal illness. We list assumptions for pathogen-specific per-case DALY weighting, population susceptibility, and risk of illness following infection in Table 1 and present an overview of dose-response calculations below.

**Table 1:** Overview of assumptions used in calculating DALYs averted, adapted from Table 7.6, *Guidelines for Drinking-water Quality*, 4^th^ Edition [24].

	Units	Probability density function (pdf) for independent variables	Values (independent variables) or formulae (dependent variables)
Untreated water quality (C_R_)	Mean organisms per litre	Log-normal (mean = standard deviation)	Means of 0.0001, , 0.001, 0.01, 0.1, or 1
Log_10_ reduction	Unitless	Uniform between bound specified	1–2, 2.01–3, or 3.01–6
Drinking-water quality (C_D_)	Organisms per litre		C_R_×(1−% reduction)
Consumption of drinking-water (V)	Litres per person per day	Mean (point estimate)	1, 2, or 5
Adherence or consistency of drinking water treatment (A)	Percent of water consumed that is treated (%)	Uniform between bound specified	50–70%, 71–90%, or 91–100%
Exposure by drinking-water (D)	Organisms per day ingested	-	Equation (1)
Risk of infection (P_inf,d_)	Per day	-	Equations (2, 3)
Risk of infection (P_inf,y_)	Per year	-	Equation (4)
Risk of diarrhoeal illness given infection (P_ill|inf_)		Mean (point estimate)	*Campylobacter*: 0.3, rotavirus: 0.5, *Cryptosporidium*: 0.7
Risk of diarrhoeal illness (P_ill_)	Per year	-	Equation (5)
Disease burden (DB)	DALYs per case	Uniform between bound specified (for rotavirus only)	*Campylobacter*: 4.6×10^−3^, rotavirus: 0.014–0.48, *Cryptosporidium*: 1.5×10^−3^
Susceptible fraction (S)	Percentage of population	Mean (point estimate)	*Campylobacter*: 100%, rotavirus: 6%, *Cryptosporidium*: 100%
Disease burden per 100,000 persons	DALYs per year per 100,000	-	Equation (6)

### Water quality assumptions

The use of QMRA models to predict reductions in waterborne disease risk requires estimation of pre- and post-treatment concentrations of microbes for which we have dose-response models. Faecal-oral pathogen occurrence and distribution are variable in drinking water sources and depend on many factors, including infection (and shedding) rates of specific pathogens in a given population, sanitation and hygiene conditions that may affect excreta containment, zoonotic reservoirs, seasonality and weather events, and other context-specific variables. No sufficiently detailed, country-level or international data exist for waterborne pathogens that would permit the meaningful estimation of means to be used in a generalizable and scientifically credible risk assessment model for drinking water risk. We have used assumptions for microbial counts in pre-treatment waters over a range from 0.0001 per litre (“low risk”) to 1 per litre (“high risk”). For the purposes of this modelling exercise, we have assigned a log-normal probability density function to water quality assumptions, which is one distribution that has been observed to characterize microbial density in water [28], [29].

### Treatment effectiveness

We modelled DALYs averted for improved drinking water quality under several different assumptions about microbiological effectiveness, including: (1), a hypothetical “best case” technology that would be able to reduce bacteria, viruses, and protozoa by 3.01–6 log_10_ (99.9%–99.9999%), typical of the most advanced single or multiple-barrier technologies for household water treatment [30], [31], [32] and consistent with well operated conventional or advanced water treatment; (2), a mid-range treatment option that could reduce all classes of microbes by 2.01–3 log_10_ (99%–99.9%), in the range of basic drinking water treatment and consistent with effective HWT options; and (3), a basic level of protection corresponding to poorly performing water treatment systems, systems without adequate disinfection, or some single-barrier HWT options (1–2 log_10_ reduction, or 90%–99%) [25]. In the probabilistic model, we assumed a uniform probability density function between the upper and lower log_10_ bounds we specify.

### Adherence

For safer water to translate into health gains, the percentage of treated water that is consumed is an important factor. For piped-in, domestic water service, this might be called *consistency of treatment*. In household water treatment, this may be called *adherence* and sometimes *compliance*. For the purposes of this modelling exercise, we use adherence and define it as the percentage of water an individual uses that is treated (0%–100%). We have divided adherence into *high* (91%–100%), *medium* (71%–90%), and *low* (50%–70%), with an assumed uniform probability density function describing the likelihood of values within this range in the probabilistic model. We assume here that the balance of the drinking water consumed by the user is untreated water. In reality, individuals and families may rely on a number of drinking water sources and treatment options, and these options may be a function of location, season, preferences, beliefs, knowledge, convenience, infrastructure, cost, previous household investment (e.g., a well or rainwater storage), or other factors. Our simplified assumption is that users have two options: treated water and untreated water that result in microbial risks that vary according to the assumptions we have specified.

### Model structure

We used Oracle Crystal Ball, Fusion Edition (release 11.1.2.1.000, www.oracle.com) to build a stochastic (Monte Carlo) model based on probability density functions (pdfs) specified for key high-leverage variables of interest, while conserving point estimates for other variables in the base model [25] and using a single-hit version for sensitivity analysis. Model output was collected following 10,000 simulations of each model configuration and descriptive statistics were computed in Crystal Ball. Model predictive and structural validity were tested using both the single-hit and probabilistic models using a structured comparison of model output across a range of default assumptions.

Table 1 describes the basic model structure. Pre-treatment water quality is assumed with means of 0.0001–1 per litre, distributed log-normally (C_R_). Based on effectiveness of treatment in reducing reference pathogens, treated drinking-water quality is calculated (C_R_). We then used assumed consumption of water per day (V) and adherence or consistency of water treatment (A) to calculate the predicted exposure by drinking-water (d) as the number of microbes ingested per day.
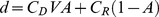
(1)We used the exponential (*Cryptosporidium*) and the Beta-Poisson (rotavirus, *Campylobacter*) models to estimate the risk of infection from exposure to microbial pathogens [26]. We calculated the risk of infection of a single microbe (d) and then calculated the annual risk from ingestion of multiple microbes over the period of a year.

The exponential model is:
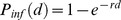
(2)Where *P(d)* is the probability of an individual becoming infected after ingesting a dose (d) and r is an infectivity constant. The value of r is 0.2 for *Cryptosporidium* (Table 1) [25].

The equation for the Beta-Poisson model is:
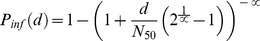
(3)N_50_ is the median infective dose, which has been experimentally derived as the dose estimated to cause infection in 50% of individuals exposed, and α is a dimensionless infectivity constant. The values for N_50_ and α are 896 and 0.145 for *Campylobacter*, respectively, and 6.17 and 0.253 for rotavirus [25], [26].

Yearly infection risk is then calculated as:
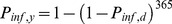
(4)


The risk of illness is calculated as:
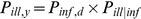
(5)where the probability of illness given infection (*P_ill|inf_*) is 0.3 for *Campylobacter*, 0.5 for rotavirus, and 0.7 for *Cryptosporidium* [24].

To calculate the disease burden in DALYs associated with yearly risk of illness, we used the following formula for each reference microbe:
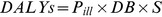
(6)Where DB is the estimated per-case disease burden in DALYs and S is the susceptible fraction of the population for the outcome of interest. Per-case DALY weights are 4.6×10^−3^ (campylobacteriosis), 0.014–0.48 (rotavirus), and 1.5×10^−3^ (cryptosporidiosis). The model further assumes that 100% of the population is susceptible to cryptosporidiosis and campylobacteriosis, but that only 6% is susceptible to illness associated with rotavirus infection (Table 1). These DALYs weights and estimates for susceptible fraction are those used in the base model as described in the WHO *Guidelines for Drinking-water Quality* [25]. To calculate the estimated disease burden among 100,000 person-years across all three reference pathogens, we multiplied the microbe-specific disease burden by 100,000 and summed estimates for *Campylobacter*, *Cryptosporidium*, and rotavirus. Figures for averted DALYs were then calculated as the difference between DALYs before and after treatment, assuming that the only alternative to treated water was untreated source water.

We conducted a four-way sensitivity analysis to determine the relative sensitivity of mean DALYs averted to the key independent variables of background water quality, litres consumed per day, treatment effectiveness, and consistency/adherence. For the sensitivity analysis, we used the single-hit model using means for all inputs to examine variability in output resulting from changes to independent variables of interest.

## Results

Predicted health impacts associated with water quality interventions under pre-treatment water quality, technology effectiveness, and adherence assumptions are presented in Table 2 as DALYs averted per 100,000 people per year. For some combinations of parameters resulting in high impact (moderate pre-treatment water quality, medium to high treatment effectiveness, and high adherence), point estimates for mean DALYs averted per 100,000 person-years are over 500. With lower levels of adherence, DALYs averted decrease dramatically when untreated water is of high risk, since adherence is of greater importance when untreated water is a source of disease. The range of estimates of effect when pre-treatment water is of moderate to high risk indicate that the potential health gains are very high as adherence approaches 100% (up to 1840 DALYs per 100,000 per year).

**Table 2:** Probabilistic model output with estimated mean DALYs averted per 100,000 persons pear year as a function of pre-treatment water quality; treatment effectiveness against bacteria, viruses, and protozoa; and adherence.

Pre-treatment water quality (log-normal distribution)	Treatment effectiveness: bacteria, viruses, and protozoa	Adherence, percentage of total water consumed that is treated	Mean DALYs averted per 100,000 persons (standard deviation)	Range	Mean standard error
**High risk:**	High	High (91–100%)	196 (178)	3.54–1,570	1.78
reference	(3.01–6 log_10_)	Medium (71–90%)	50.4 (28.7)	0–523	0.29
pathogens		Low (50–70%)	21.5 (16.8)	0–129	0.17
present,	Medium	High (91–100%)	173 (140)	0–1,740	1.40
mean:	(2.01–3 log_10_)	Medium (71–90%)	49.2 (28.2)	0–494	0.28
1 per litre		Low (50–70%)	20.8 (16.7)	0–86.0	0.17
	Low	High (91–100%)	104 (62.0)	0–1,010	0.62
	(1–2 log_10_)	Medium (71–90%)	43.3 (26.1)	0–347	0.26
		Low (50–70%)	19.4 (15.9)	0–85.8	0.16
**Moderate**	High	High (91–100%)	520 (326)	29.5–1,840	3.26
**high risk:**	(3.01–6 log_10_)	Medium (71–90%)	174 (149)	4.91–1,130	1.49
reference		Low (50–70%)	68.1 (62.6)	3.95–659	0.63
pathogens	Medium	High (91–100%)	498 (316)	36.0–1,890	3.16
present,	(2.01–3 log_10_)	Medium (71–90%)	170 (146)	6.82 (1,110)	1.46
mean:		Low (50–70%)	69.0 (63.5)	1.14–676)	0.63
0.1 per litre	Low	High (91–100%)	363 (257)	20.0–1,460	2.57
	(1–2 log_10_)	Medium (71–90%)	147 (127)	5.41–998)	1.27
		Low (50–70%)	63.7 (57.6)	2.93–604	0.58
**Moderate**	High	High (91–100%)	533 (313)	26.9–1,630	3.13
**risk:**	(3.01–6 log_10_)	Medium (71–90%)	369 (213)	19.3–1,180	2.13
reference		Low (50–70%)	216 (131)	6.56–682	1.31
pathogens	Medium	High (91–100%)	527 (304)	31.4–1,650	3.04
present,	(2.01–3 log_10_)	Medium (71–90%)	365 (211)	18.6–1,140	2.11
mean:		Low (50–70%)	215 (129)	6.69–724	1.29
0.01 per litre	Low	High (91–100%)	483 (277)	24.5–1,520	2.77
	(1–2 log_10_)	Medium (71–90%)	338 (196)	17.9–1,140	1.96
		Low (50–70%)	206 (124)	8.23–676	1.24
**Moderate**	High	High (91–100%)	133 (127)	2.15–1,100	1.27
**low risk:**	(3.01–6 log_10_)	Medium (71–90%)	109 (102)	3.14–966	1.02
reference		Low (50–70%)	78.5 (71.3)	2.29–557	0.71
pathogens	Medium	High (91–100%)	133 (125)	3.49–1,180	1.25
present,	(2.01–3 log_10_)	Medium (71–90%)	109 (101)	2.80–865	1.01
mean:		Low (50–70%)	79.3 (70.5)	2.20–615	0.71
0.001 per	Low	High (91–100%)	129 (121)	2.06–1,070	1.21
litre	(1–2 log_10_)	Medium (71–90%)	105 (98.6)	2.55–885	0.99
		Low (50–70%)	74.9 (67.0)	1.82–511	0.67
**Low risk:**	High	High (91–100%)	18.3 (15.9)	0–231	0.18
reference	(3.01–6 log_10_)	Medium (71–90%)	15.5 (13.3)	0–231	0.15
pathogens		Low (50–70%)	11.5 (9.92)	0–212	0.12
present,	Medium	High (91–100%)	19.3 (15.8)	0–546	0.19
mean:	(2.01–3 log_10_)	Medium (71–90%)	16.1 (13.3)	0–311	0.16
0.0001 per		Low (50–70%)	11.9 (9.94)	0–221	0.12
litre	Low	High (91–100%)	18.5 (15.5)	0–319	0.18
	(1–2 log_10_)	Medium (71–90%)	15.2 (12.9)	0–377	0.15
		Low (50–70%)	11.3 (9.60)	0–157	0.11

Results suggest that when pre-treatment water is of moderate to high risk, adherence is more important than treatment effectiveness. To take one example from Table 2, when pre-treatment water is of moderate high risk, the mean estimated DALYs averted per 100,000 person-years for a technology with low effectiveness (1–2 log_10_ reduction of bacteria, viruses, and protozoa) but that is used very consistently (91–100% adherence) is 363, compared with 68.1 for a technology that is more effective (3.01–6 log_10_ reduction of bacteria, viruses, and protozoa) but that is used less consistently (50–70% adherence).

Assuming lower risk untreated water, both the potential health gains from water treatment and the sensitivity to adherence are reduced, suggesting that the importance of water treatment and high adherence are elevated in high-risk waters and reduced in low-risk waters. We used a uniform probability density function in the model to indicate “high” adherence between 91%–100% (individual simulation estimates being equally likely to be assigned a value between these in individual simulations), a range which probably represents highest likely achievable adherence *in situ*.

**Figure 1:**
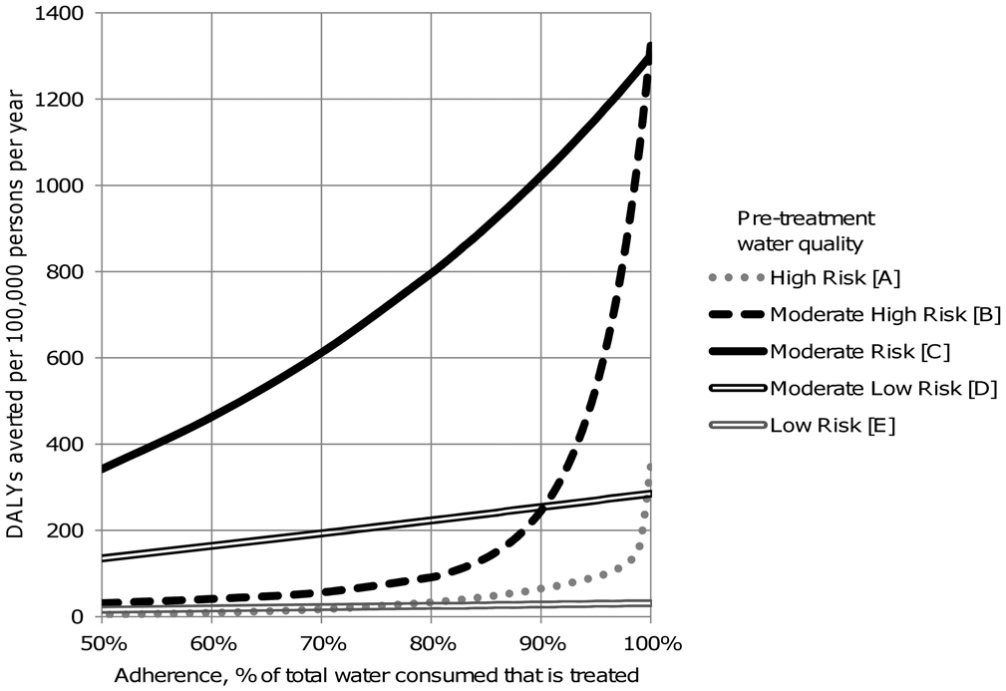
DALYs averted per 100,000 population, per year, based on assumptions about technology effectiveness (2 log_10_ reduction in each pathogen class), adherence, and background water quality assumptions from **Table 2**.

Figure 1 presents a graphical illustration of how the predicted health gains vary as a function of both adherence and pre-treatment water quality. Calculation of DALYs averted in Figure 1 assumed a 2 log_10_ reduction across all reference pathogens and 1 litre per day consumption. When pre-treatment water was of high risk [A] and [B], there is a dramatic decline in predicted health gains with decreasing adherence. Water that is of moderate risk [C] is less affected by reduced adherence, although sharp declines are observed. Waters that are already low risk [D] and [E] do not show a strong association between DALYs averted and adherence because the risk of drinking untreated water is already low.

Results of the sensitivity analysis (Table 3) indicate that model output is highly sensitive to pre-treatment water quality and adherence, moderately sensitive over the range of assumptions to treatment effectiveness against waterborne pathogens, and relatively insensitive to volume consumed per person per day over realistic values (1–5 litres per day per person). A straightforward comparison of point estimates across changes in water quality, adherence, effectiveness and consumption suggest that, within pre-treatment water risk categories, DALYs averted estimates are most sensitive to adherence. Reducing adherence from 100% to 90% reduces the number of DALYs averted by over 90% under some model assumptions and up to 96%, with sharper declines when pre-treatment water quality is higher risk (Table 3 and Figure 1). The model was also very sensitive to the per-case DALY disease burden estimates (data not shown), which have been derived elsewhere and are key assumptions included in the base model we used [25], [26] (Table 1).

**Table 3:** Sensitivity analysis across a range of pre-treatment microbial counts of reference pathogens and adherence, at “high” and “low” effectiveness (6 log_10_ and 2 log_10_, respectively) and at 2 levels of water consumption (1 litre per person per day and 5 litres per person per day).

Consumption, litres per person per day	Treatment effectiveness: bacteria, viruses, and protozoa	Adherence, percentage of total water consumed that is treated	DALYs averted per 100,000 people per year over pre-treatment water risk categories				
			High: 1 per litre	Moderate high: 0.1 per litre	Moderate: 0.01 per litre	Moderate low: 0.001 per litre	Low: 0.0001 per litre
1	6 log_10_ (99.9999%)	100%	1680	1618	1339	290	32
		90%	69	278	1049	258	29
		80%	35	96	815	227	25
		70%	18	57	626	196	22
		60%	9	41	473	166	19
		50%	5	31	349	137	16
1	2 log_10_ (99%)	100%	347	1324	1302	285	31
		90%	65	245	1022	254	28
		80%	33	91	796	223	25
		70%	17	56	612	193	22
		60%	9	41	463	164	19
		50%	5	31	342	135	16
5	6 log_10_ (99.9999%)	100%	1679	1612	1333	288	32
		90%	69	278	1045	257	28
		80%	35	96	812	226	25
		70%	18	57	624	195	22
		60%	9	41	471	166	19
		50%	5	31	348	136	16
5	2 log_10_ (99%)	100%	100	626	1181	272	30
		90%	50	157	934	243	27
		80%	26	75	732	213	24
		70%	14	50	567	185	21
		60%	7	38	432	157	18
		50%	4	29	321	129	15

Reported means are from single-hit models.

## Discussion

When and where water is an important source of pathogen exposure, water quality interventions can reduce exposure to pathogens and result in improved health. Our results suggest, however, that the potential health gains are reduced sharply with even occasional consumption of untreated drinking water.

Our results are consistent with the findings of a similar QMRA analysis by Hunter et al. (2009) [6]. This study concluded that the health benefits of improved quality drinking water (as delivered by centralised treatment and distribution) were limited if even a small percentage of overall water consumed was of lower quality, for example during interruptions of service in piped water supply of when the alternative drinking-water source was surface water. Hunter *et al.* concluded as we have that the overall risk attributable to drinking water is controlled by those periods of higher exposure risk when no quality protection is in place, reducing overall impacts of water quality improvements significantly if the intervention is not present a high percentage of the time. We propose that our results are applicable to water quality interventions generally, including centralized water treatment.

There is reason to believe, however, that there is even greater risk among the estimated 1.2 billion who report treating water in the home. First, most of those that practice HWT reside in low-income settings where the risk of highly contaminated water is greatest. Second, unlike residents of higher-income settings who may receive warning when their water treatment systems fail, these individuals are unable to judge the quality of their drinking water and must follow the practice daily. Third, those who consistently treat their water at home are likely to be exposed to untreated drinking water when they are away from the home—a fundamental shortcoming of HWT compared to effective community-wide improvements in water quality.

Reconciling these results with the existing evidence base for water quality interventions raises certain questions. Our findings suggest that the dramatic protective effects against diarrhoeal disease reported in recent systematic reviews of household water treatment [14], [15], [16], [17] and other water quality interventions [33], [34] would seem to be unlikely in the absence of high adherence. Unfortunately, even studies that report on coverage and overall use rarely report on the extent to which participants consume untreated water. Nevertheless, our findings are consistent with two recent studies of the same water filter among young children in low-income settings. While investigators found no protective effect from an intervention in which most participants acknowledged regularly drinking untreated water [19], the intervention was highly protective against diarrhoea when few participants consumed untreated water (Rachel Peletz, personal communication).

Another potential explanation is that the large protective effects reported by open trials of water quality interventions are exaggerated due to reporting bias [15], [18]. Correct, consistent use has been an ongoing challenge for HWT, and few studies appear to reach the levels of uptake that our results suggest would be necessary to achieve the reported effects on diarrhoea. In this respect, our results lend support to the assertion that published health impact estimates of water quality interventions may have been overestimated. An analysis of reported DALYs averted by HWT interventions and a comparison with this and other modelling efforts may be a useful next step in contextualizing the evidence base for water quality interventions.

Another possibility that may explain higher than expected estimates of health effects from trials with low apparent adherence is that water quality interventions may have been more likely to have been used consistently when untreated water was perceived to be of higher risk due to aesthetic or other indicators, and those perceptions may have correlated with actual levels of pathogens (i.e., that adherence is higher when it is more important, when water is of higher risk). More research is needed on whether and how HWT behaviours are driven by perceptions of water safety, and whether and how perceptions of water safety are related to actual water safety.

Our results may also help explain the diminished health impact that has been observed from longer-term evaluations of HWT interventions [14], [16]. A number of studies of HWT have shown reduced use of HWT interventions over time, raising questions about the potential for sustained use [19], [20], [21], [22], [23], [24]. Technologies with a high user burden, recurrent costs, or those that involve substantial behaviour change may be especially challenging to achieve the continued high adherence that our results indicate is necessary to sustain health impact [35]. Boiling has overcome many of these same obstacles and become the leading HWT method worldwide [36]. However, studies of the effectiveness of boiling demonstrate the challenge of achieving consistently safe drinking water even from this highly normative practice.

Comparing predicted health gains resulting from two levels of microbiological treatment effectiveness (2 log_10_ and 6 log_10_ reduction of reference pathogens) suggests decreasing marginal health gains with increasing efficiency (Table 3). A focus on increasing microbiological effectiveness of water quality interventions, for example from 99% to 99.9999%, may not result in proportionately lower risk, especially if adherence will be less than 100%; the swamping effect of lower than 100% adherence essentially makes the real differences between microbial reduction efficiencies meaningless with respect to outcomes as predicted in this modelling exercise. We acknowledge that the value of requiring higher efficiencies for water treatment technologies is due to the higher efficiencies that may be required under outbreak scenarios, when untreated water may be of higher risk, or if there are no alternatives to high risk source waters. Given the fact that some technologies may be adopted more readily and used more consistently than other technologies (even those that may be more effective in reducing microbes), these results would seem to suggest that a focus on promoting adherence may be more critical in delivering health gains than focusing exclusively on increasing microbiological effectiveness [31].

The health impact estimates we report here should be interpreted in light of the uncertainty of the assumptions and necessary simplifications used to produce them. Pre-treatment water quality, for example, is likely to be highly variable in any given setting. Dose-response models for reference pathogens have been derived from few studies, mostly using data from healthy adults in wealthier countries. As an approach to estimating health risk, QMRA incorporates a number of assumptions whose values and ranges are uncertain. Where possible, we have attempted to use assumptions that would tend to result in conservative estimates of health impacts, and realistic ranges of pre-treatment water quality, adherence, treatment effectiveness in reducing microbes, and other key variables. QMRA is a quickly evolving approach and models like the one we have used will benefit from further refinement of methods and assumptions.

The greatest risks of waterborne disease globally are from microbial pathogens, although chemical contaminants are locally significant risks to public health. This risk assessment does not address the potential problem of anthropogenic or naturally occurring radiological or chemical contaminants that may be present in drinking-water, including but not limited to pesticides, arsenic, fluoride, heavy metals, nitrate, excess salts, disinfection by-products, pharmaceuticals, or others. Our study also omits helminthic and other diseases that may be transmitted by drinking water. Including these additional factors would require additional data and different modelling approaches.

Current guidelines supporting HWT emphasize the need for correct, consistent and sustained use [31] and our results underscore the need to make high adherence an essential priority in HWT design and program implementation. In order to maximise health gains from interventions, it is necessary to provide HWT solutions that people will use exclusively or nearly so. This may require not only intensified behaviour change interventions, but also process improvements in HWT products and technologies that minimize the likelihood of non-adherence. In order to monitor and evaluate these and other water quality interventions, it is also important to develop and improve tools for monitoring and to ensure that field studies report adherence. Given the challenges of achieving this high level of adherence through point-of-use water treatment, these results also confirm the need to continue efforts to provide safe, reliable, piped-in water to household taps in order to realize the full promise of health gains from water quality interventions.
